# Physicians’ knowledge, attitudes and practices on advance care planning for patients with advanced cancer in a National University Hospital in the Philippines

**DOI:** 10.3332/ecancer.2024.1725

**Published:** 2024-07-03

**Authors:** Sachiko S Estreller, John Christopher Pilapil, Frederic Ivan Ting, Irisyl Real, Edward Christopher Dee

**Affiliations:** 1 Department of Medicine, University of the Philippines, Philippine General Hospital, Manila 1000, Philippines; 2 Section of Medical Oncology, Department of Internal Medicine, Corazon Locsin Montelibano Memorial Regional Hospital, Bacolod 6100, Philippines; 3 Department of Clinical Sciences, College of Medicine, University of St. La Salle, Bacolod 6100, Philippines; 4 Division of Medical Oncology, Department of Medicine, University of the Philippines, Philippine General Hospital, Manila 1000, Philippines; 5 Division of Radiation Oncology, Memorial Sloan Kettering Cancer Center, New York, NY 10065, USA

**Keywords:** physicians, knowledge, attitudes, practices, advanced care planning, Philippines

## Abstract

**Purpose:**

Advance care planning (ACP) is generally part of patients’ rights and decision-making processes. It is a component of the patient–physician care dynamics, especially in the context of life-threatening illness. Little is known about ACP and the utilisation of advance directives in the Philippines, a country of 110 million people. The study aimed to explore the knowledge, attitudes and practices (KAPs) of resident physicians in a national university hospital in the Philippines regarding ACP for patients with advanced cancer.

**Methods:**

Using a cross-sectional design involving resident physicians, an online survey with a self-administered questionnaire was distributed and answered by a total of 202 respondents.

**Results:**

Results show that resident physicians generally: (1) view palliative and hospice medicine to be the same and without differences, (2) are comfortable with discussing ACP and prognosis of medical conditions with patients and their families, but (3) do not regularly initiate or offer ACP to them, (4) identify a lack of time, fear of imparting emotional distress to patients and their families and personal discomfort as barriers to conducting ACP and (5) have had no formal training for ACP but are willing to undergo such formation, given the opportunity.

**Conclusion:**

This study highlights the continuing need to bridge and unite KAPs pertaining to ACP among physicians. Further studies should be undertaken to device a proper training program and better explore the complexities of end-of-life care as it is experienced by Filipino patients with advanced cancer.

## Introduction

We live in an era of longevity. Advances in medicine have led to longer lifespans and an increase in people living with chronic diseases, including cancer. With added years of life also comes the possibility of prolonged suffering and increased healthcare burden for patients and caregivers alike [1]. This has highlighted the relevance of in-depth knowledge of the concepts of supportive care in any field of medicine.

Palliative care seeks to improve the quality of life of patients diagnosed with life-threatening illnesses and receiving curative treatment through the timely initiation of supportive interventions [2]. This is in contrast to hospice care wherein the focus is shifted from curative to comprehensive comfort care [3].

American Society of Clinical Oncology (ASCO) guidelines recommend that patients with advanced cancer should be referred to interdisciplinary palliative care teams early in the disease trajectory, alongside active cancer treatment. The optimal time for initiation of palliative care is not yet well-defined, but the current recommendation is to refer patients within 8 weeks of the diagnosis of advanced cancer [4].

Advance care planning (ACP) is part of palliative and hospice care. It is intended for patients who wish to ensure that they receive interventions consistent with their beliefs and priorities during serious and chronic illness [5]. ACP is a product not only of the individual, but of the institutional culture the patients are in, the knowledge and attitudes of those involved in their care and the manner in which end-of-life discussions are carried out [6, 7]. ACP leads to better patient experience at the end of life. It was found to reduce the number of hospitalisations and utilisation of cardiopulmonary resuscitation, increase the use of hospice care and increase the frequency of out-of-hospital care for terminal patients [6].

Palliative and hospice care in the Philippines began in the 1980s and is a growing field of medicine in the country. While its provision is still insufficient due to lack of awareness, limited palliative care workforce and high out-of-pocket health care costs, palliative and hospice care hold importance in a growing population living with chronic diseases such as cancer [8, 9]. 

However, there are many issues surrounding ACP in the Philippines – questions of who is responsible for initiating ACP, the optimal time for discussions, barriers and facilitators and how it should be carried out for patients to ensure effective ACP [7]. Physicians themselves may sometimes find it difficult to initiate conversations about ACP because they lack self-efficacy in their skill set [10].

There is a paucity of data exploring ACP in the Philippines. This study aimed to explore the knowledge, attitudes and practices (KAPs) of resident physicians in a national university hospital in the Philippines with regard to ACP for patients with advanced cancer.

## Materials and methods

### Study design and study setting

The cross-sectional study was implemented at the University of the Philippines, Philippine General Hospital from October to November 2020. It was conducted as a cross-sectional online survey, with a self-administered questionnaire answered by the participants.

### Study population

The sample size was set at 195 residents, computed based on a two-tailed 95% level of confidence, a power arbitrarily set at 80%; an estimated number of residents eligible for inclusion at 294, arbitrarily set at 50% due to lack of available information regarding the KAPs of resident physicians in the country regarding advanced care planning. An oversampling of 10% was also considered to account for the variability of the study population, non-response or incomplete information. Stratified, systematic and random sampling was performed with proportional allocation across the different hospital departments [11].

### Inclusion and exclusion criteria

The study included all resident physicians of the Philippine General Hospital, regardless of year level, who managed and cared for adult patients with cancer. Respondents from the following departments were included: Medicine, Surgery, Neurosciences, Family and Community Medicine, Obstetrics and Gynecology, Orthopedics, Ophthalmology and Otorhinolaryngology.

Resident physicians from departments that did not involve directly caring for cancer patients were excluded, namely the departments of: Anesthesiology, Radiology, Rehabilitation Medicine, Emergency Medicine and Pathology.

### Research instruments

The questionnaire utilised (Appendix B) was based on the published study of Snyder, Allen and Radwany published in 2012, entitled ‘Physician Knowledge, Attitude and Experience with ACP, Palliative Care, and Hospice’ [12]. Permission to use the original English version and modify the questionnaire (to add demographic data of the residents such as age, sex, religion, residency training program and year level) from the primary author was secured, after which, was reviewed by palliative care and medical oncology consultants from our institution for face and content validation before use in this study.

### Data collection

The survey was distributed through an online platform (Google Forms) for data collection. Informed consent was taken and access to the self-administered questionnaire was subsequently given. A reminder to fill out the questionnaire was sent to the participants on the day of distribution, after 3 days and after a week.

### Data processing and analysis

The data were extracted and encoded onto an electronic spreadsheet file. The subsequent data processing and analysis were then carried out using the statistical software, Stata 13. Descriptive statistics were used and data were presented using tables and pie charts to illustrate the distribution of responses from study participants.

### Ethics

Ethics approval was obtained before the conduct of this study from the University of the Philippines Manila Research Ethics Board (UPMREB-2020-366-01).

## Results

### Baseline characteristics

A total of 202 residents were recruited with a response rate of 76% (202 out of 265). The target sample size was achieved for the Departments of Family Medicine (24), Surgery (33), Internal Medicine (56), Obstetrics and Gynecology (44) and Orthopedics (13). Involved participants were short of the established number for the Departments of Neurosciences (12), Ophthalmology (10) and Otorhinolaryngology (10).

Survey respondents had a mean age of 28 years old. Of the 202 recruited, 45% were male and 55% were female. The majority of the respondents were Catholic with those identifying as Muslim comprising the smallest subpopulation. Most of the respondents were in their 1st and 2nd years of training. On average, the study population had been practicing medicine for 2.6 years. Notably, 86% have never had any training in ACP. The respondents who received training in ACP were mainly from the Department of Family and Community Medicine (57%). Table 1 summarizes the baseline characteristics of the resident physicians involved in the study.

### Knowledge on palliative care and hospice

Most physicians were aware that palliative and hospice care were not the same. A large proportion (78%) were likewise aware that hospice care is a subdiscipline of palliative care. The majority of the participants (72%) were cognizant that palliative care is not confined to patients who only have less than 6 months to live. Table A1 (in Appendix A) summarizes the distribution of ratings given by resident participants in terms of their knowledge of palliative care and hospice.

### Attitudes towards advance care planning

Knowledge of patients’ end-of-life directives was important for the majority of the respondents (65%), being either in agreement or strong agreement with the statement. Many respondents (62%) were comfortable discussing ACP with patients, with a slightly lower proportion (54%) being comfortable with discussing patients’ prognosis. While most answered otherwise, a substantial proportion (32%) of the physicians in the study believe that ACP takes too much of their time with a patient. Meanwhile, 44% believe that ACP is too upsetting for patients and their families. Table A3 (in Appendix A) shows the responses and ratings of physician attitudes towards ACP.

### Practices in advance care planning

**Figure 1.**
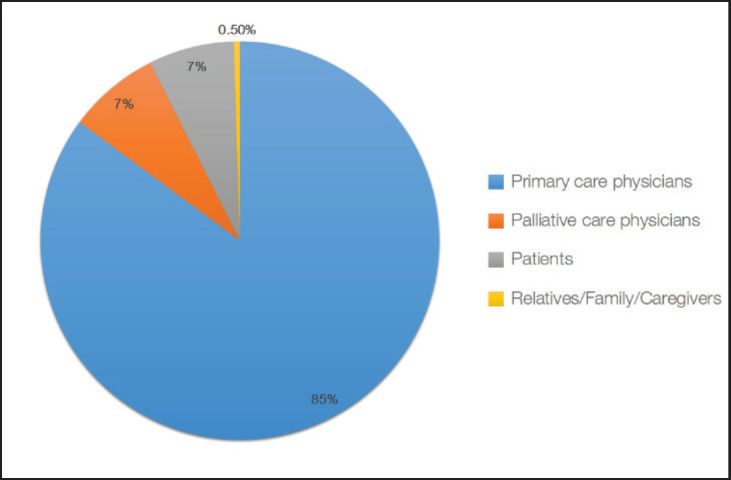
Initiators of ACP in clinical practice.

In the clinical practice of our respondents, most (85%) believed that primary care physicians are responsible for initiating ACP discussions. The relatives, family and/or caregivers are perceived to be the least responsible (1%) for initiating ACP discussions (Figure 1).

**Figure 2.**
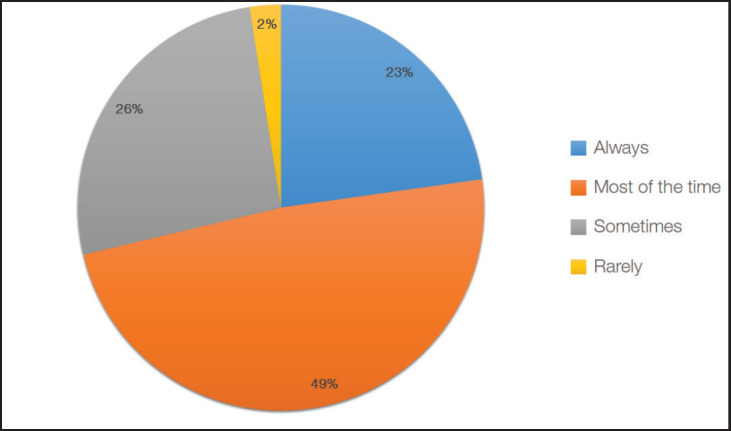
Frequency of offering ACP in clinical practice.

Almost half (49%) of the resident physicians would offer ACP to their patients most of the time but only 23% are consistent in always offering such services and directives (Figure 2).

**Figure 3.**
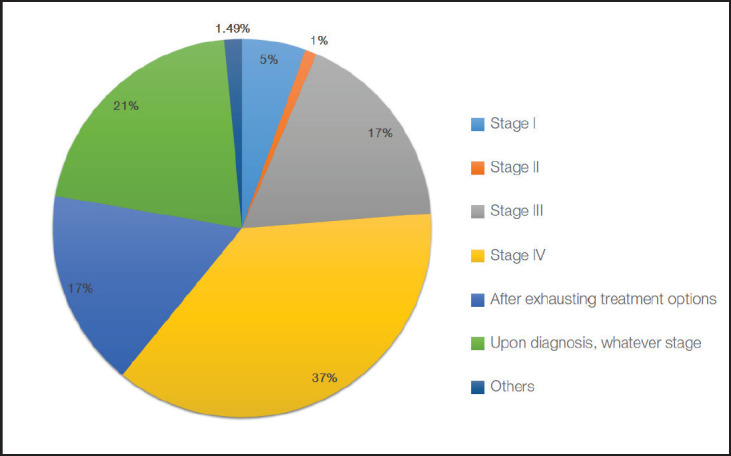
Stage of cancer and clinical triggers for initiating ACP.

Regarding the timing of initiating ACP discussions, 37% of our participants offer ACP to patients with stage IV cancer, while some respondents offer ACP at earlier stages of the disease: Stage I (17%), Stage II (21%) and Stage III (17%). Of note, 21% opt to offer ACP and the creation of advance directives (ADs) for cancer patients upon diagnosis, regardless of stage. Only 1.5% initiate ACP discussions at a much later phase in patient care, after all treatment options are exhausted (Figure 3).

**Figure 4.**
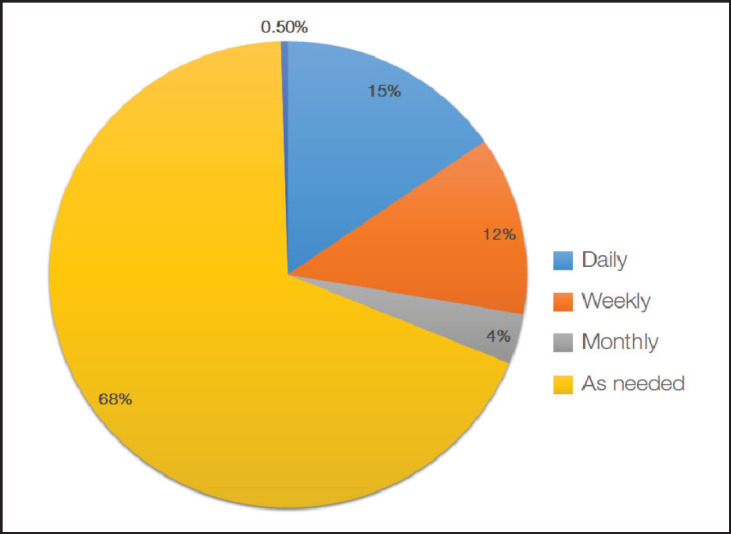
Timing of revisions and modifications of ACP.

Most (68%) of the respondents reviewed and revised decisions made through ACP on an as needed basis. There are progressively less who do so on a daily, weekly and monthly basis. A minority (0.5%) of respondents do not revisit ACP directives (Figure 4).

The relatives of patients are involved in ACP discussions most of the time. The decisions arrived upon are carried out most of the time in the practice of half of the physician respondents. ACP directives are properly and legally documented, but personal family lawyers (which is not a requirement in the hospital setting in the Philippines since in-house legal counsel exists) are not involved in the majority.

Patients are usually perceived by their physicians to be satisfied by the conduction of ACP most of the time. A similar trend is seen with the relatives of the patients.

### Willingness to learn more about advance care planning

**Figure 5.**
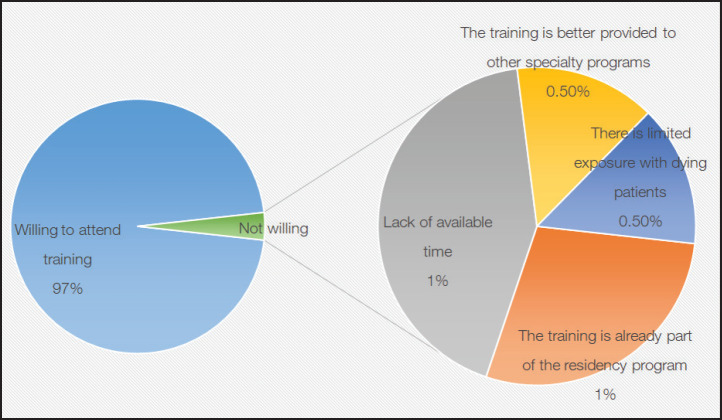
Willingness of participants to learn more about advance care planning and reasons cited for unwillingness.

Almost all (97%) respondents were willing to attend a training course for ACP. Among those who were not willing, the most frequently cited reasons were lack of available time and that training was already a part of their residency program (Figure 5).

## Discussion

There is a need for studies exploring palliative care, hospice and ACP specifically in the Philippine setting since cultural intricacies pose threats and opportunities to effective delivery of end-of-life care. This study explored the KAPs of physicians in a national university hospital who care for advanced cancer patients.

The concepts of palliative and hospice care are often interchanged despite their differences in focus and interventions. Palliative care is applicable early in the course of illness and includes interventions with curative intent, while hospice care focuses on giving peace, comfort and dignity to the dying and their families [13]. In this study, although most physicians recognised the difference, almost a quarter (23%) still have the misconception that palliative and hospice care are the same. This gap in knowledge may lead to delayed referrals of patients. Physicians’ competence in explaining hospice and palliative care can potentially lead to its better integration into patient management [14, 15]. It is important for physicians to explain that initiation of ACP does not equate to surrendering to the diagnosed disease but rather an exercise of good foresight that benefits both patients and their families/caregivers alike.

Results of our study showed inconsistencies in attitudes and practices related to ACP among respondents. More than half were willing and comfortable in facilitating ACP discussions. However, only 23% reported that they always offered ACP to their patients in practice. This inconsistency between attitude and practice was also seen in similar studies that attributed it to differences in culture and communication, lack of knowledge, confusion of roles and past negative experiences of physicians with ACP [16, 17]. 

Another inconsistency between attitude and practices that our study found was in the timing of ACP initiation. While most participants acknowledged that palliative care is not limited to patients with short life expectancy, the majority of respondents offered ACP later in the disease trajectory or even after all attempts at treatment have been exhausted. These findings are consistent with other studies that found most physicians initiated ACP when patients were already unwell, had a major decline in functional status, or when treatment options were already exhausted [7].

A delay in ACP discussions is concerning as early palliative care has been shown to consistently improve the quality of life of cancer patients [6, 13–15]. Patients who discussed their prognosis and plans with their physicians in a timely manner are thought to be more likely to make better medical and personal decisions, to be less distressed and more emotionally prepared for death [18, 19].

Our study also investigated the factors that influence ACP as it is implemented in real-life practice. Identified barriers to ACP included lack of time, fear of giving emotional distress and discomfort in disclosing disease prognosis. These barriers are similar to other studies done that showed limited time and emotional distress were associated with delays in ACP discussions [20, 21]. Religious fatalism and the strong family orientation of Filipinos also preclude ACP discussions for fear of bringing about more distress [18]. Physicians’ apprehension in predicting prognosis and applying population statistics for individual patients is also a barrier to ACP [22, 23]. 

ACP is a collaborative process respecting not only the patients’ autonomy, but also ‘relational autonomy’ which is heavily influenced by social and emotional factors from their families and physicians [10]. In this study, we found that most physicians involved patients’ relatives in ACP discussions. Taking a patient-centered approach with family involvement is key to successful ACP and has been found to increase the quality of life and reduce the incidence of depression and anxiety among patients and family members [24–26].

Involvement of family members, however, is a double-edged sword. In our study, 18% of respondents noted that the advance care wishes of patients were not honored. This can perhaps be partially attributed to the power of the relatives’ wishes to override patients’ directives [27]. In the context of the family-driven culture of Filipinos, this points to the physicians’ role in maintaining open lines of communication while conducting ACP to set goals that are clear and concordant between patients and their families [21].

ACP involves the formulation of an AD, usually in the form of a written document signed by the patient and/or their families. In this study, we found that most ADs are put into formal writing (73.3%), but a personal family lawyer is often not involved since the hospital has its own legal counsel that takes charge of these matters. Better quality of life in patients has been associated with ADs that reflect their own goals and wishes [28–30].

ACP utilisation is positively correlated to physicians’ experiences and specialisation [20, 21], with oncologists being more accepting of their role in initiating ACP discussions compared to other disciplines, likely because ACP competency is integrated into their training. Most of the respondents in our study did not have any form of training in ACP, but almost all have the willingness to attend one. In addition, the majority of respondents believe that it is their duty as primary care physicians to initiate ACP discussions with patients. These findings are hopeful: physicians from diverse fields of medicine acknowledge the importance of ACP training and appear eager to gain this valuable clinical skill.

Physicians with training on ACP, those who have less discomfort talking about death and those who have a good grasp of prognosis were found to be more supportive of palliative care referral at all stages of disease and initiate end-of-life discussions in a timelier manner [31]. Effective ACP with AD heavily relies on physicians who can facilitate prognostic understanding through good communication skills [31, 32]. It is, therefore, important for physicians from all specialties to be trained in ACP for improved delivery of end-of-life care.

Among those who received training, we found that most of these residents came from the Department of Family and Community Medicine. Strengthening the existing training program for these residents and possibly extending it to other specialty departments would be one way to improve the practice of ACP in our hospital.

Limitations of this study include the following: (1) sample sizes were not met for three departments, although the overall sample size was large; (2) only residents were included, while fellows and consultants were excluded; and (3) results were presented without subgroup analysis per department or specialisation.

## Conclusion and recommendations

ACP is a complex process involving social and cultural factors at play between physicians, patients and their families. There has been a general lack of interest and investment in implementing national initiatives related to advanced care planning, a relatively new concept of care in the Philippines. Thus, our study serves as an impetus to integrate ACP into the healthcare system in our country.

By describing the KAPs towards ACP, this study highlighted physicians’ perceived barriers to effective ACP such as lack of time, concerns about emotional distress and discomfort with prognostication. This study also identified physicians’ perceived strengths to effective ACP such as the utilisation of written directives, involvement of patients and family members in ACP and willingness of physicians for further ACP training.

Further studies to describe ACP from the perspective of the patient and their families are recommended, and this information, together with the findings of our study, can lead to better understanding and improved delivery of ACP in a clear, timely and collaborative manner.

## Conflicts of interest

The authors of this paper declare no conflicts of interest in the conduction of the study.

## Funding

Dr Dee is funded in part through the NIH/NCI Support Grant P30 CA008748 outside the submitted work.

## Figures and Tables

**Table 1.** Baseline characteristics of the population.

Variables	Summary measures (%)
Age in years	28.55 ± 2.26
Sex	
Male	92 (46%)
Female	110 (54)
Religion	
Catholic	160 (79)
Christian	32 (16)
Islam	1 (1)
Agnostic/Non-practicing	9 (4)
Year level	
First year	66 (33)
Second year	73 (36)
Third year	42 (21)
Fourth year	17 (8)
Fifth to Sixth year	4 (2)
Years of medical practice	2.57 ± 1.46 (0-10)
Residency program	
Family medicine	24 (12)
Surgery	33 (16)
Internal medicine	56 (28)
Neurology	12 (6)
Obstetrics & gynecology	44 (22)
Ophthalmology	10 (5)
Orthopedics	13 (6)
Otorhinolaryngology	10 (5)
Training in advanced care planning	
Yes	28 (14)
No	174 (86)

## Appendix A: Other tables

**Table A1.** Distribution of ratings in terms of knowledge.

Responses	Strongly disagree	Disagree	Neutral	Agree	Strongly agree	NA
Palliative care and hospice are virtually the same.	48 (23.76%)	79 (39.11%)	28 (13.86%)	37 (18.32%)	9 (4.46%)	1 (0.50%)
Hospice is a form of palliative care, but not all forms/branches of palliative care are part of hospice care.	2 (0.99%)	17 (8.42%)	9 (4.46%)	79 (39.11%)	79 (39.11%)	16 (7.92%)
Palliative care is appropriate for patients only when they have less than 6 months to live.	76 (37.62%)	70 (34.65%)	21 (10.40%)	13 (6.44%)	11 (5.45%)	11 (5.45%)

**Table A2.** Distribution of ratings in terms of practices.

Responses	Never	Rarely	Sometimes	Most of the time	Always	NA
How often are a patient’s relatives involved in your discussions with them regarding ACP?	-	3 (1.49%)	21 (10.40%)	95 (47.03%)	73 (36.14%)	10 (4.95%)
How often are decisions of your patients and/or their relatives/caregivers regarding ACP carried out properly and fully?	-	6 (2.97%)	42 (20.79%)	100 (49.50%)	46 (22.77%)	8 (3.96%)
How often are decisions made by your patients and/or their relatives/caregivers regarding ACP and ADs put into formal writing or legal documentation?	1 (0.50%)	12 (5.94%)	33 (16.34%)	90 (44.55%)	58 (28.71%)	8 (3.96%)
How often is a lawyer/attorney involved in discussions and decisions of your patients and/or their relatives/caregivers regarding ACP and ADs?	114 (56.44%)	52 (25.74%)	11 (5.45%)	15 (7.43%)	4 (1.98%)	6 (2.97%)
How often do you think that your patients are satisfied by the manner by which ACP and ADs creation was done for them?	-	9 (4.46%)	79 (39.11%)	95 (47.03%)	15 (7.43%)	4 (1.98%)
How often do you think that the relatives/caregivers of your patients are satisfied by the manner by which ACP and ADs creation was done for them?	-	7 (3.47%)	79 (39.11%)	94 (46.53%)	19 (9.41%)	3 (1.49%)

**Table A3.** Distribution of ratings in terms of attitudes.

Responses	Strongly disagree	Disagree	Neutral	Agree	Strongly agree	NA
Knowledge of patients’ wishes for their goals of care/end of life directives is important for all medical practitioners.	2 (0.99%)	-	1 (0.50%)	13 (6.44%)	119 (58.91%)	67 (33.17%)
In general, I am comfortable having ACP discussions with my patients	7 (3.47%)	20 (9.90%)	34 (16.83%)	65 (32.18%)	62 (30.69%)	14 (6.93%)
It takes too much of my time to discuss ACP with a patient.	24 (11.88%)	62 (30.69%)	48 (23.76%)	51 (25.25%)	14 (6.93%)	3 (1.49%)
I feel comfortable communicating to patients the prognosis of their medical condition(s).	8 (3.96%)	33 (16.34%)	48 (23.76%)	72 (35.64%)	36 (17.82%)	5 (2.48%)
Advance care wishes are rarely honored.	36 (17.82%)	84 (41.58%)	42 (20.79%)	29 (14.36%)	10 (4.95%)	1 (0.50%)
ACP is too upsetting for patients and their families	6 (2.97%)	55 (27.23%)	52 (25.74%)	65 (32.18%)	24 (11.88%)	-

## Appendix B: Questionnaire

**RESPONDENT’S PROFILE**

**Name (optional):** __________

**Age: __________ Sex: M / F**

**Religion: __________**

**Residency Training Program: __________**

**Year Level: __________**

**Years of Medical Practice: __________**

**Have you had any training in ACP? Yes / No**

**PART 1.**

To what extent do you agree or disagree with the following statements? Please encircle the number corresponding to your answer:

- 1 – Strongly disagree
- 2 – Agree
- 3 – Neutral
- 4 – Agree
- 5 – Strongly agree
- N/A – Not applicable

**Table:**

Palliative care and hospice are virtually the same.	1	2	3	4	5	N/A
Hospice is a form of palliative care, but not all forms/branches of palliative care are part of hospice care.	1	2	3	4	5	N/A
Palliative care is appropriate for patients only when they have less than 6 months to live.	1	2	3	4	5	N/A
4. Knowledge of patients’ wishes for their goals of care/end of life directives is important for all medical practitioners.	1	2	3	4	5	N/A
5. In general, I am comfortable having ACP discussions with my patients	1	2	3	4	5	N/A
6. It takes too much of my time to discuss ACP with a patient.	1	2	3	4	5	N/A
7. I feel comfortable communicating to patients the prognosis of their medical condition(s).	1	2	3	4	5	N/A
8. Advance care wishes are rarely honored.	1	2	3	4	5	N/A
9. ACP is too upsetting for patients and their families	1	2	3	4	5	N/A

**PART 2.**

How often do you observe the following in your practice as a physician treating/managing cancer patients? Please encircle the number corresponding to your answer.

- 1 – Never
- 2 – Rarely
- 3 – Sometimes
- 4 – Most of the Time
- 5 – Always
- N/A – Not applicable

**Table:**

10. How often are a patient’s relatives involved in your discussions with them regarding ACP?	1	2	3	4	5	N/A
11. How often are decisions of your patients and/or their relatives/caregivers regarding ACP carried out properly and fully?	1	2	3	4	5	N/A
12. How often are decisions made by your patients and/or their relatives/caregivers regarding ACP and ADs put into formal writing or legal documentation?	1	2	3	4	5	N/A
13. How often is a lawyer/attorney involved in discussions and decisions of your patients and/or their relatives/caregivers regarding ACP and ADs?	1	2	3	4	5	N/A
14. How often do you think that your patients are satisfied by the manner by which ACP and ADs creation was done for them?	1	2	3	4	5	N/A
15. How often do you think that the relatives/caregivers of your patients are satisfied by the manner by which ACP and ADs creation was done for them?	1	2	3	4	5	N/A

**PART 3.**

Encircle the letter of your answer to the following questions:

16. Who is primarily responsible for initiating ACP discussions?

- Primary physicians
- Social workers
- Palliative care physicians
- Attorneys
- Patients
- Patient’s relatives/family members/care givers
- Others, please specify: _________________

17. Among your patients diagnosed with cancer, how often will you offer ACP and the creation of ADs?

- Always
- Most of the time
- Sometimes
- Rarely
- Never

18. At what stage of cancer diagnosis will you begin to offer ACP and the creation of ADs?

- Stage I
- Stage II
- Stage III
- Stage IV
- Only after all treatment options have been exhausted
- Upon diagnosis, regardless of stage
- Others, please specify: __________

19. How often would you review and ask for possible modifications and/or revisions on ADs previously given by a patient and/or the patient’s relatives/caregivers?

- Daily
- Weekly
- Monthly
- Whenever a change in the disease course or prognosis occurs
- Never

20. If given the opportunity, would you be willing to take a preparatory basic course for ACP?

- Yes
- No. If no, please specify reason: __________
